# Evaluating the network adequacy of vision care services for children in Arizona: A cross sectional study

**DOI:** 10.3934/publichealth.2024007

**Published:** 2024-01-29

**Authors:** Rizwana Biviji, Nikita Vora, Nalani Thomas, Daniel Sheridan, Cindy M. Reynolds, Faith Kyaruzi, Swapna Reddy

**Affiliations:** 1 College of Health Solutions, Arizona State University, Phoenix, AZ; 2 College of Arts and Sciences, Emory University, Atlanta, GA; 3 School of Medicine, University of Arizona, Phoenix, AZ

**Keywords:** health disparities, access to care, vision care, pediatric, network adequacy

## Abstract

**Background:**

Vision challenges are among the most prevalent disabling conditions in childhood, affecting up to 28% of school-age children. These issues can impact the development, learning, and literacy skills of affected children. While vision problems are correctable with timely diagnosis and treatment, insufficient networks can impede children's access to comprehensive, and high-quality care.

**Objective:**

The study aims to determine where pediatric vision care network adequacy exists in the state of Arizona and where there are gaps in receiving vision care for children.

**Methods:**

This cross-sectional study assessed the adequacy of pediatric vision care networks in Arizona through a “secret shopper” phone survey. Calls were made to practices that accept Arizona's Medicaid program, Arizona Health Care Cost Containment System (AHCCCS) and/or commercial insurance. Providers were contacted following a standardized script to schedule routine appointments on behalf of 10 and 3-year-old patients enrolled in either Medicaid or commercial health insurance plans. The study examined various components of children's access to vision care services, including the reliability of provider directory information, time until the next available appointment, bilingual service offerings, ages served, region of practice and types of care available.

**Results:**

A total of 556 practices in Arizona were evaluated through simulations as patients on AHCCCS, and 510 practices were assessed through simulations as patients with commercial health insurance plans. The average wait time for the next available appointment was 13 days for both insurance types. Alarmingly, up to 74% of vision care practices in Arizona do not serve children covered by AHCCCS. Furthermore, only 41% provide services to children 5 years and younger.

**Conclusions:**

Our findings underscore the need to improve access to vision care services for children in Arizona, especially racial/ethnic minorities, low-income groups, and rural residents.

## 1. Introduction

Transitioning towards a more comprehensive and holistic healthcare model necessitates acknowledging all dimensions of an individual's health that significantly contribute to their overall well-being and longevity. Historically, however, the American healthcare system has often treated some aspects of physical health, such as vision care, as separate entities, thereby negatively impacting patient care, access, and outcomes. The Centers for Disease Control and Prevention (CDC) recommends vision screenings for children at various intervals: that a child should be screened at between newborn to 3 months, 6 months to 1 year, 3 years, and 5 years [1]. As children enter school age, regular eye exams every two years are recommended [2]. Common vision problems among children aged 3 to 5 include misaligned eyes (strabismus), lazy eye (amblyopia), and refractive errors such as myopia, hyperopia, and astigmatism, as well as other focusing problems [3]. Among children, vision challenges that are not appropriately addressed can significantly impact a child's ability to learn, meet appropriate educational benchmarks, and engage in peer interactions. Moreover, such challenges may result in partial or permanent vision loss [4]. Additionally, unaddressed vision challenges are associated with lower early literacy performance and pre-reading skills during preschool and kindergarten, serving as strong predictors of overall school performance throughout a child's K-12 education [5],[6]. As many as 28% of school-age children experience vision problems that can affect their development, learning, and literacy skills [6]. Additionally, one in twenty preschool age children faces vision challenges, yet only 39% have had their vision checked [1],[5]. The financial implications of childhood vision issues are substantial, with reports estimating costs of up to $10 billion annually related to vision loss in children [7]. These estimates encompass various factors, including medical care, vision aids, special education, caregiving, vision screening programs, federal assistance, and overall quality of life [4],[7].

Though strides have been made to include and expand vision care through public and private health plans, disparities persist in utilization, access and outcomes, particularly affecting the most vulnerable members of our society. Nationally, disparities in vision care are evident across various factors and social determinants, including race/ethnicity, gender, age, and geography. These disparities not only impact outcomes but also highlight challenges in accessing care [8]. Black children and families earning below 400% of the federal poverty level experience twice the rate of expenditures associated with emergency vision care, posing a likely barrier to preventive and regular office centered care as needed [9]. Similarly, more than one-third of Mexican American and non-Hispanic B lack adolescents experience inadequately corrected refractive disorders [10]. Specifically, in Arizona, children face significant health disparities and inequities, with 15% residing in high poverty areas, well above the national average of 9%, as of 2019 [11]. The need for public health insurance is substantial in the state, as over 30% of the population- 2.5 million individuals, is enrolled in Medicaid, or Arizona Health Care Cost Containment System (AHCCCS) [12]. Vision services for all AHCCCS members under the age of 21 include regular eye exams and vision screenings, prescription eyeglasses, and repairs or replacements of broken or lost eyeglasses [13]. Notably, the state of Arizona only recently mandated vision screening for children in 2019, a significant policy change [14]. However, Arizona children remain less likely to have their vision tested within the past two years. In the age range of 0–5, only 31.4% of Arizona children have received a recent vision screening, compared to the national average of 38.9% [6].

Inadequate networks can prevent pediatric patients from accessing the vision providers they trust and depend upon. This requires not only having insurance coverage, but also sufficient access to providers listed in coverage networks. Enrolling in an insurance plan holds little value if the providers in that insurance network do not cater to children in need or offer essential options for their families to access the care they provide. Therefore, the primary aim of this study was to assess the adequacy of pediatric vision care networks (the capacity to provide local vision services for children), in Arizona and identify any gaps in vision care for children. Access to care is defined by the availability of services based on region, type of care (Optometry/Ophthalmology), accepted payment methods (insurance), age groups served, and languages in which services are offered.

## 2. Study data and methods

### 2.1. Source of data

This cross-sectional study was designed to test pediatric vision care network adequacy in the state of Arizona via a “secret shopper” phone survey conducted through calls to practices accepting AHCCCS and/or commercial health insurance. There are three main providers of vision care: ophthalmologists, optometrists, and opticians [15]. Ophthalmologists are either Doctor of Medicine (MD) or Doctors of Osteopathy (OD) who are qualified to give comprehensive vision care, including vision services, eye exams, eye surgery, and diagnosis and treatment of vision diseases or complications. Optometrists are considered Doctor of Optometry, which means that they can examine both internal and external eye structures. However, the main difference between optometrists and ophthalmologists is that optometrists are not trained to perform surgery or to manage all eye diseases. Lastly, opticians are healthcare professionals who are specialists in dispensing and fitting of glasses. The dataset of practicing pediatric vision care providers in Arizona, i.e., ophthalmologist (MD and DO) and optometrist (OD) was built using existing databases from the Arizona Medical Board, Arizona Board of Optometry and Arizona Osteopathic Board. The raw data consisted of 56178 MD, 12 DO, and 1323 OD (included data on expired/canceled licenses, out of state practices, and other specialties). These databases were further cleaned to include only those providers who are active, specializing in ophthalmology or optometry and licensed in the state of Arizona.

**Figure 1.**
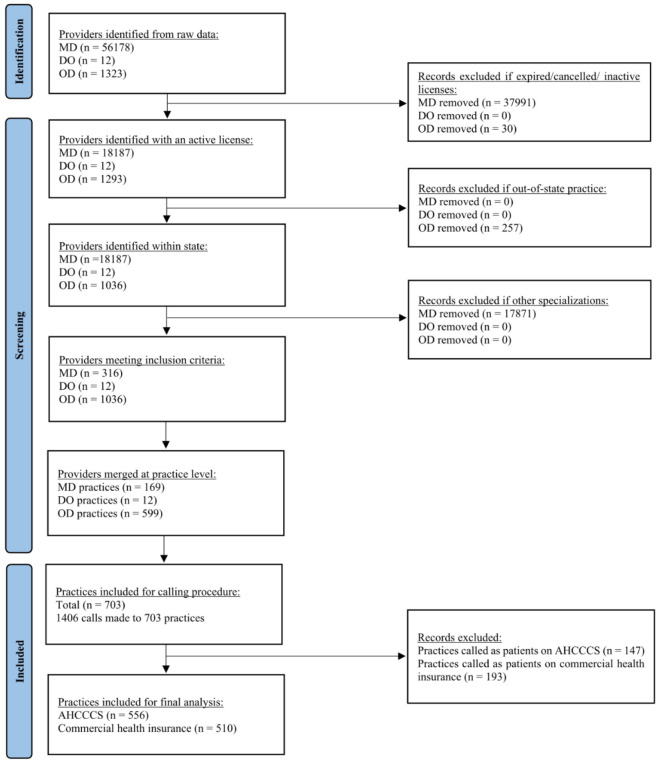
Flowchart detailing the sample selection process.

For the Doctor of Medicine (MD) data set, we identified a total of 18187 physicians who were listed as “in state” and were either “active”, “active with restrictions”, or “active with limitations”. Next, we included only those providers who specialized in ophthalmology or pediatric ophthalmology with a listed address in the state of Arizona. From there, we organized the data to consolidate physicians who worked in the same practice to one address. This process was repeated three times, resulting in a total of 316 physicians at 169 different practices (Figure 1). The data reflects licensed providers as of March 2022.

For the Doctor of Osteopathy (DO) dataset, the most recent provider list from the year 2019 received from the licensing board included 12 active DOs at 12 different practices in the state of Arizona specializing in ophthalmology. The optometrist (OD) dataset from the Arizona Board of optometry included a total of 1293 active ODs. From this we identified a total of 1077 ODs with listed addresses in the state of Arizona. However, a large number of providers recorded their residential addresses under the mailing address designation in the database. To accurately categorize providers licensed and practicing in the state of Arizona, we conducted an additional step where each provider name was manually entered into Google by three reviewers. If the initial Google search results did not produce an optometrist, the OD's name, followed by “optometry” was inputted to refine the results. This step helped us identify each provider's official mailing address and practice location. Next, we sorted the providers by practice location to categorize the data at practice level (similar to MD dataset), which resulted in a total of 1036 ODs at 599 practices (Figure 1). The data reflects licensed providers as of March 2022. Additionally, the three datasets were further collapsed to identify a consolidated list of practices across all three provider types which resulted in a total of 703 unique practices in the state of Arizona. The Arizona State University (ASU) Institutional Review Board (IRB) approved and deemed this study as exempt.

### 2.2. Study design

To adequately test the network adequacy of Arizona's pediatric vision care provider network, we contacted each individual practice (*n* = 703) using a “secret shopper” phone survey. The phone survey tested various components of children's access to needed vision care through a standardized script of questions, including reliability of provider directory information, appointment availability at the practice level for children enrolled in AHCCCS and those with commercial health insurance, language access, and compliance with regulatory standards. The variables of interest were: (i) time until the next available appointment, (ii) time of day for appointment, (iii) after hours and weekend appointment availability, (iv) if the practice was reached, (v) if the practice was accepting new patients, (vi) if the practice accepts a specific insurance plan, (vii) if the practice offers online booking options, (viii) if the practice offers bilingual services, (ix) region of practice, (x) ages served and (xi) if the patient needs referral from a primary care provider to be seen.

We contacted providers following a standardized script (Supplementary) as part of the secret shopper methodology to schedule a routine appointment posing as parents of 10 and 3-year-old patients enrolled in either AHCCCS or commercial health plan.

A “secret shopper” study approach similar to that used here is one in which researchers simulate a potential patient seeking care to better understand the actual parameters and patient experience parameters in an area of health care delivery. A primary strength of this approach is that it provides valuable insight into the access related barriers that are difficult to measure through other investigative methods [16]. Existing literature supports the use of this research methodology especially for programs such as Medicaid (AHCCCS) as an ethical means of testing the compliance of public programs with government-enforced regulatory standards [17]–[21]. By using this approach, rather than customer surveys or structured interview phone calls, data can be collected more cost effectively, as well as more efficiently. Furthermore, the use of secret shoppers eliminates the “Hawthorne effect” also known as the way individuals alter their behavior when they are aware they are being observed [19]. The study therefore mimics a real-world setting when a patient is trying to schedule an appointment with a provider, effectively testing if the provider in fact offers care within the networks that they hold out to serve, while also examining the aforementioned layers of access. We found that the secret shopper methodology allowed us to collect sufficient data, even with limited resources.

AHCCCS contracts with seven integrated managed care organizations (MCOs) across three Geographic Service Areas (GSAs) in the state (Central, North, and South). For the purpose of portraying simulated patients for our survey, the most prominent MCO in terms of membership was chosen within each of the 3 GSAs- Central GSA: Mercy Care; North GSA: Care 1st; South GSA: Banner University Family Care. Blue Cross Blue Shield of Arizona and Avesis were the two commercial insurers simulated in the calls placed to MD/DOs and ODs respectively.

### 2.3. Survey instrument

The standardized script (Supplementary) used for the phone survey in this study was adapted from the works of Steinman et al. (2012) and Reddy et al. (2021), which was used to test network adequacies for pediatric psychiatric services and pediatric oral health services respectively [18],[19]. The script included questions about scheduling an appointment with a vision care provider (appointment date, time of appointment, evening and weekend appointment availability), along with questions about needing a referral from another provider or scheduling an appointment with another clinician, e.g., optometrist (OD) first before seeing an ophthalmologist (MD/DO). In addition, we included questions concerning patient intake (accepting new patients, accepting specific insurance plans, and ages served). And, if unable to schedule an appointment, information of another provider practice. We also included questions on the languages in which services were offered and whether the practice allowed for online appointment scheduling. An online scheduling system is a web-based application or portal that allows enrollees to conveniently book their appointments through a web-enabled device. Further, to capture the most comprehensive information from each call, detailed field notes were recorded during our conversations with the scheduling staff. These field notes were a summary of the key takeaway points from our conversations with the office personnel.

To test the validity of the survey instrument, two research assistants made 100 phone calls each posing as a parent with AHCCCS and one with commercial health insurance. To ensure a natural flow of conversation and to glean maximum information, the script was modified during the process based on caller experience and the specific needs of the study. Moreover, in instances where the office requested identifying information, the researchers clarified that they were not prepared to schedule an appointment at the time but were mainly interested in gathering information about the wait time and scheduling procedures.

### 2.4. Calling procedure

Five research assistants trained to pose as secret shoppers made 1406 calls to 703 practices between May 2022 to June 2022 (Figure 1). Each provider practice received one call from a parent of a 10 and 3-year-old child enrolled in AHCCCS and one call from a parent of a 10 and 3-year-old child enrolled in a commercial health insurance plan wanting to schedule a routine eye exam. For each call, research assistants documented in the directory whether the practice could be reached. If the researcher was not able to reach the practice upon first call, they attempted to call again a week later for a maximum of two additional attempts after which the practice was excluded from further analysis. Calls to the same practice on behalf of a commercially insured and AHCCCS-insured patient were conducted one week apart and at different times to encounter different office staff members who fielded the calls. This was to ensure that the staff member does not recognize the script from previous calls. All calls were conducted during business hours from 9 am to 12 pm and 1 pm to 5 pm to allow for lunch break.

Through the data collection process, we identified additional practices that were excluded from further analysis. The reasons for exclusion were: (i) could not reach after three attempts, (ii) inactive provider, (iii) calls truncated due to requested identifying information, (iv) surgical centers and specialty centers, (v) invalid phone number or business address. This resulted in a total of 556 practices (79.09% of those cataloged) included on the AHCCCS side and 510 practices (72.55% of those cataloged) on the commercial health insurance side. The number of providers included for further analysis was reduced to a total of 1194.

## 3. Data analysis

### 3.1. Quantitative analysis

The data collected through the secret shopper survey were compiled and analyzed. The outcome variable time until the next available appointment (wait time) was based on the number of days one had to wait for their appointment. This was calculated by subtracting the date of the appointment from the date when the call was made. The variable time of appointment was categorized into morning (before 12 pm), afternoon (before 5 pm) and evening (after 5 pm) based on the hour of appointment in the day.

First, descriptive statistics (mean, range, frequency) were calculated for the following metrics: time until next appointment, time of day for appointment, after hours and weekend appointment availability, acceptance of new patients, acceptance of specific insurance, online booking options, bilingual service offerings, type of provider, need referral from other provider, and ages served. Next, an independent *T*-test was used to study the mean difference in appointment wait time at the practice level for children covered under AHCCCS versus commercial health insurance. Statistical significance was assessed at the *p* < 0.05 level.

### 3.2. Qualitative analysis

Field notes from our phone survey were analyzed using a general inductive content analysis approach [22],[23]. In this approach, themes were derived from data, as opposed to using preconceived categories [24]. A total of 1,066 field notes recorded from calls made on behalf of AHCCCS and commercial health insurance holders were included in final analysis. First, two coders [RB and SR] undertook an independent reading of a random sample of 100 field notes to establish consistency in the textual unit of analysis, identification of categories, and formation of themes [25]. Next, to assess coding consistency, five reviewers independently analyzed a new sample of 100 field notes [25]. Agreement among coders was high. The five coders independently coded the remaining sample and met regularly to resolve any coding discrepancies and discuss the themes that were detected in the data. This process resulted in 11 initial themes that discussed the barriers or challenges of accessing pediatric vision care services. These themes were further condensed to classify similar sub-themes into one category, which resulted in 3 major thematic categories.

## 4. Study results

### 4.1. Access to care

**Figure 2.**
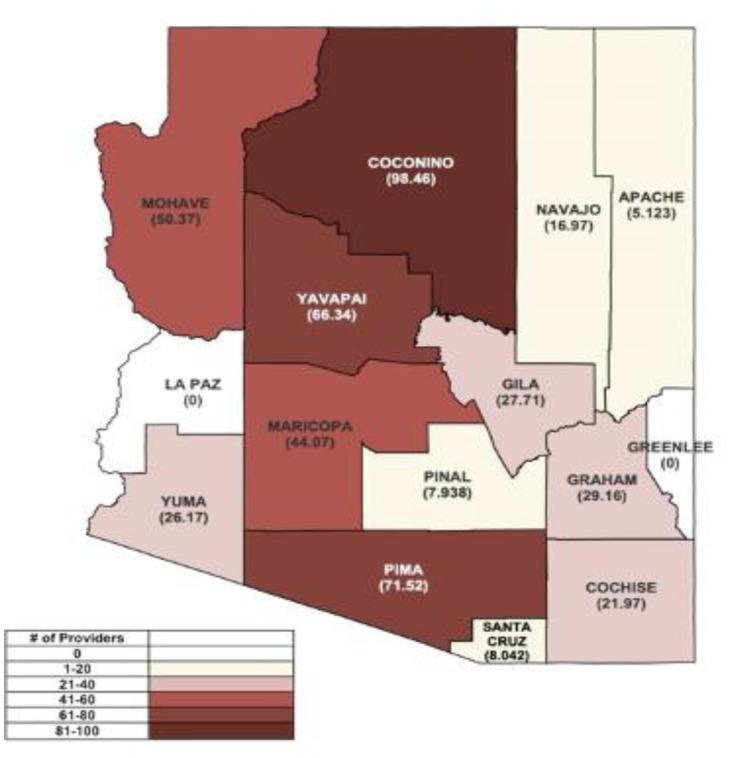
Number of pediatric eye care providers (accepting patients 18-years-old and younger) per capita (100,000) per county in Arizona*. Note: *The number of providers who accept pediatric patients 18-years-old and younger was divided by the population of children 18-years-old and younger for each county.

The state of Arizona is predominantly rural, with several counties designated as medically underserved areas (MUA) by the Arizona Department of Health Services. The MUA designation is given to counties with limited access to primary care services and primary care physicians (PCPs) [26]. As such, we observed a higher concentration of providers in more urban counties, particularly Maricopa (*n* = 831, 69.6%) and Pima (*n* = 217, 18.2%). In fact, in only 5 out of 15 Arizona counties (Coconino, Maricopa, Mohave, Pima, Yavapai) (Figure 2), the concentration of pediatric eye care providers accepting patients 18 years old and younger per capita in a 100,000 population exceeds 40.

Approximately two-thirds of providers in the state, or 61.3%, serve children aged 18 and younger. More specifically, 41.0% of providers in the state serve pediatric patients 5 and younger. In fact, some providers even offer services to children as young as 6 months old. However, those who specialize in young children (<5 y/o) have limited availability, often practicing at specific times and locations. In addition, the pediatric ophthalmologists and other providers serving this population generally have long waitlists, thereby creating additional barriers in seeking prompt care.

*“We see patients as young as 3-years-old, but the provider is only in the office for routine eye exams once a month.”*

*“The provider sees patients under 6 on Monday, Tuesday, and Wednesday mornings only. Plus, it will be a longer wait to see him.”*

Certain practices have very specific criteria for the young patient to be seen by the provider. This would include the patient's ability to read the alphabet, recognize the shapes, know their numbers, or to be able to sit still for the routine eye exam.

*“So long as the patient can recognize the alphabet, we will see them.”*

Some providers indicated serving very young patients (<3 y/o), but they exclusively accept medical insurance and do not take vision insurance, such as Avesis. On the other hand, some providers do not accept any insurance for routine eye exams. Furthermore, a minority of practices (27.0%) accommodate pediatric patients starting at various ages (e.g., >6 years, >7 years, >8 years, >10 years) depending on the facility's capability to deliver the required care. Reasons cited for this variation included considerations like appropriate equipment fitting, provider expertise, or the patient's ability to communicate, answer questions or tolerate eye dilation. Additionally, many practices directed us to other providers who specialize in treating very young children.

*“We accept appointments for children 10 years and older. You have to get your child's eyes dilated by another provider at a different facility in order to have the eye exam.”*

15.8% of remaining practices exclusively cater to adults ages 18 and older. Notably, some practices refrain from providing routine eye exams for pediatric patients but extend their services to address medical issues and specialized care.

### 4.2. Patient intake

Ensuring a seamless patient intake is vital when scheduling appointments. Factors discussed here include a practice's availability to accept new patients, compatibility with the patient's insurance type, provision of online scheduling options, offering services in languages other than English, and the ability to accept patients with a referral from their PCPs. Table 1 provides an overview and contrast of caller experiences by insurance type.

**Table 1.** Variability in patient intake experiences by insurance type.

Patient Intake	AHCCCS	BCBSAZ/Avesis
Number of practices identified	556	510
Practices accepting new pediatric patients	78.0%	90.4%
Insurance accepted	25.7%	69.1%
Provider referral needed	8.7%	1.8%
Online scheduling available	42.9%	56.4%
Bilingual services available	45.8%	58.9%

While the majority of listed practices were open to new patients, a higher percentage of practices informed the commercial health insurance caller (90.4%) about their acceptance of new patients compared to the AHCCCS caller (78.0%). Reasons cited for not accepting new patients include provider unavailability, specialization exclusively in medical conditions, or a focus that excludes pediatric patients.

*“Our physician retired sooner than expected with a wait list going into August or September 2022.”*

*“Usually a Neurology specialist sees patients here, however, the OD comes in occasionally to do routine appointments.”*

Up to 69% of practices accepted the specified commercial health insurance plans (Blue Cross Blue Shield of Arizona or Avesis), while only 26% accepted AHCCCS. Within the practices accepting AHCCCS, 1.3% exclusively accepted specific AHCCCS plans, while others restricted AHCCCS to medical conditions only. Notably, two practices did not accept AHCCCS but provided free routine eye exams. In several instances, the practice explicitly declined to accept the specified insurance. This trend was particularly noticeable among AHCCCS holders compared to those with commercial health insurance.

*“Our facility is medically contracted with AHCCCS, but not visually.”*

In fact, a receptionist explicitly requested that we refrain from mentioning our AHCCCS coverage, stating that they could not schedule an appointment if we disclosed it.

The majority of practices did not require referrals from a PCP for routine eye exams. Only 8.7% and 1.8% of practices informed the AHCCCS caller and commercial health insurance caller, respectively, of the need for a PCP referral. These practices specified that the patient could pay out of pocket for an eye exam, ranging from $50 to $550. Additional tests could incur higher costs, with some reaching up to $400.

*“The visit must be deemed medically necessary in order to be covered by insurance.”*

*“For Blue Cross Blue Shield of Arizona, if there is an NNJ in front of the insurance number, we need a referral from a primary care physician.”*

Other practices reported accepting AHCCCS for specific services only, with additional services or products incurring an out of pocket fee.

*“We do not accept AHCCCS and the cash payment for a routine eye exam would be roughly $100 depending if dilation or extra exam were needed.”*

*“AHCCCS will cover routine eye exams, but not lenses or glasses. It will not cover additional tests such as refraction which costs $45 USD.”*

One of the national optical chain practices stated that they do not accept AHCCCS as they are not considered a “provider”. Additionally, a few providers mentioned being out of network for the specified commercial health insurance plan.

*“We just stopped being in-network for Avesis but are willing to offer 50% off from the out-of-pocket fee of $95.”*

We also observed that a few practices do not specify the accepted commercial health insurance on their online website, while others incorrectly listed that they accept Avesis. Furthermore, some practices do not handle direct billing to insurance; instead, they provide patients with invoices for the services rendered, placing the responsibility on patients to navigate insurance reimbursements themselves.

Around 56.0% practices informed the commercial health insurance caller that they have online scheduling options available, while 43.0% conveyed this information to the AHCCCS caller. Additionally, as high as 58.9% of practices reported offering bilingual services to the commercial health insurance caller, compared to 45.8% for the AHCCCS caller. Among those offering bilingual services, the majority provided services in Spanish, with only a minority of practices extending services to other global languages (American Sign Language, Bosnian, Burmese, Croatian, Hindi, Konkani, Korean, Mandarin, Marathi, Portuguese, Punjabi, Serbian, Tamil, Telugu, Turkish, Urdu, Vietnamese).

As a highly diverse state, Arizona presents unique needs concerning the languages spoken for communication with healthcare providers. The state comprises a substantial Hispanic population, and patients often find greater comfort communicating in Spanish with their care team. From our observations, certain limitations and challenges exist on the provider's end. For instance, not all providers are bilingual, leading to potential delays when scheduling with a bilingual provider or staff member. Additionally, some providers offer bilingual services only on specific days of the week (e.g., Monday or Thursday only). This setup may pose significant access barriers, particularly for children attending school or for parents/caregivers who are working professionals and unavailable during those specific days and times.

*“All of our optometrists are not bilingual, but we do have a few techs that are available to speak Spanish.”*

*“Wait time for a bilingual provider is a lot longer. Ends up being 2 weeks away.”*

We observed that the scheduling staff were often accommodating of our need to converse in a second language and attempted to provide alternative options. One staff member suggested the option to ‘FaceTime’ with a relative or friend who could speak both languages during the appointment. Some mentioned having technologies in place for translation needs (e.g., phone translating lines, translating apps on tablet/iPad, video chat with translators), while others strongly recommended bringing in our own translators.

*“Our optometrists do not speak Spanish, but we do have a way of pulling up a translator.”*

*“We have people who can translate outside the doctor's office but do not have anyone who can translate inside the doctor's office. You would have to bring your own translator into the office.”*

On the flip side, in a few instances, we encountered resistance or hesitancy from the receptionist when inquiring about bilingual offerings.

*“I mean the kid just needs to know his letters; language shouldn't matter for that.”*

Additional challenges acting as barriers to scheduling routine eye exams included encounters with rude or uncivil office staff, especially evident when the practice did not accept the specified insurance, particularly AHCCCS. In such cases, accommodating requests for bilingual providers or after-hours/weekend appointments proved difficult. Receptionists were noted to be in a hurry to ‘get off the phone’.

*“If we see you, we would be seeing you assuming you are unable to pay.”*

Some practices exclusively focus on treating medical conditions of the eye and do not offer routine eye exams. Their services may be limited to pain management, retinal issues, glaucoma treatment, plastic surgery, and other ocular conditions. Alternatively, these practices have extended wait times to see the provider.

*“We are unable to accept new patients until next year.”*

In other attempts, we were kept on hold for extended periods, such as 20 minutes in one instance, to schedule an appointment.

### 4.3. Appointment availability

**Figure 3.**
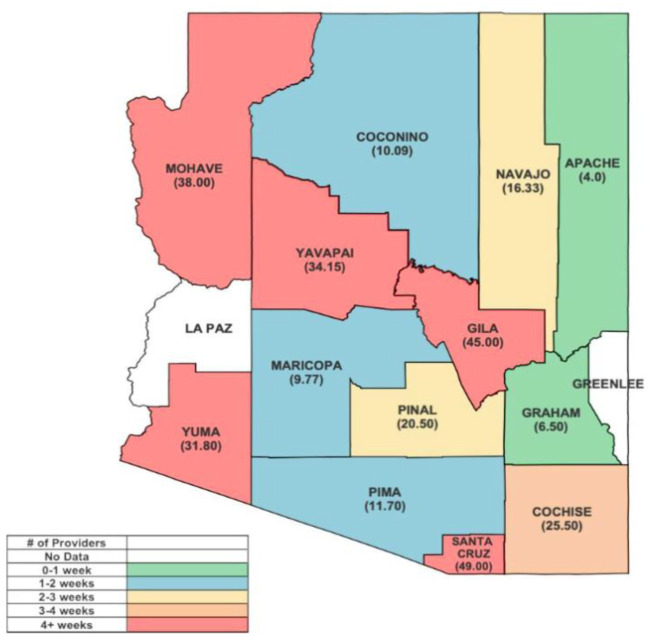
Average wait time (in days) per practice in Arizona per county*. Note: *Average wait time was calculated by the time that would pass from the time of the initial call and the first available appointment.

The average wait time for the next available appointment was 13 days, with a median of 7 days for both insurance types. No significant differences were observed in appointment wait times between callers with commercial health insurance and those insured through AHCCCS [*t* (756) = 0.275, *p* = 0.783]. For AHCCCS callers, the wait time ranged from 0 to 97 days, with at least 26 practices violating AHCCCS Contractors Manual (ACOM) Policy 417, which requires routine appointments be available within 45 days of request [27]. The wait time for commercial callers ranged from 0 to 147 days. At the county level, more urban counties of Coconino, Maricopa, and Pima had an average wait time of less than two weeks for both insurance types, while rural counties such as Gila, Mohave, Santa Cruz, Yavapai, and Yuma had average wait times exceeding 4 weeks (Figure 3).

Appointments were predominantly offered during the morning and afternoon for both insurance types. Notably, more practices informed the commercial caller (19.5%) that they had all-day availability compared to the AHCCCS caller (4.2%). However, the availability of weekend or after-hour appointments did not significantly differ across insurance types, with approximately 45% of practices offering alternate appointment schedules upon inquiry.

## 5. Discussion

This study complements existing evidence on care gaps in vision health for children within Arizona and nationally. To our knowledge, this is a first study that assesses network adequacy for vision care services in a state such as Arizona. Our findings reveal critical gaps in network adequacy that may significantly impact access to care and service utilization for children. Given that the original goal of Medicaid was to provide medical care funding for low-income individuals, with a particular emphasis on children and families, it is imperative that the state's network adequately aligns with this goal [28]. While our work identified some positives, such as a higher concentration of providers in more urban counties in Arizona, as well as the majority of practices accepting new patients across both insurance types and not requiring referrals from PCPs, our findings align with previous studies showing inconsistent offerings and services for patients enrolled in Medicaid [17]–[21]. This misalignment falls short of the program's promise and intended purpose. Fortunately, these findings highlight opportunities to enhance access to care for children's health. Based on these identified barriers, we recommend quality improvement approaches to address network adequacy for Medicaid and commercial payers.

Our study reveals critical barriers related to scheduling and provider availability for initiating care. In rural areas, fewer, if any, providers were available, with an average wait time of 13 days across both insurance types. Notably, 26 practices were in direct violation of the AHCCCS policy, which mandates that networks ensure routine appointments are available within 45 days of request. This finding aligns with previous studies documenting reduced network adequacy and provider scheduling availability for Medicaid services, especially in rural areas, as a significant barrier to care [18],[29],[30]. Additionally, disparities associated with social determinants of health, such as race/ethnicity, gender, age, and geography further underscore the validity of the causal relationship between childhood well-being and low income as a structural inequity [8],[31],[32]. Provider shortages, especially in rural counties, further exacerbate vision loss and vision care issues in existing medically underserved areas [32]. To address these challenges, we recommend the increasing provider availability through community cohesiveness, offering online scheduling, and expanding the number of providers offering services to all ages of pediatric patients to align with CDC recommended screening guidelines [1],[33],[34]. Furthermore, equipping federally qualified health centers to provide eye care services, both in Medicaid expanded and non-Medicaid expanded states, and providing education and training subsidies for vision care providers in rural communities, can significantly improve outcomes in children [1].

From a care delivery and cultural perspective, Arizona has a high Hispanic population, with 22% of the population speaking Spanish (above the national average of 17%) [35]. Amongst Spanish speakers in the state, 34.8% report speaking English “less than very well” [35]. Yet only 50% of providers were bilingual or offered bilingual staff, and were not always available for appointments which created longer wait times. Additionally, a diverse workforce is critical to provide culturally responsive care in the patient's primary language. Studies echo the benefits of patient-provider concordance as an ethical imperative because it bolsters diversity and improves patient outcomes in initiating and continuity of treatment for diverse patients [30],[36]–[38]. Increasing language services is a primary approach to address health disparities in vision care services. For example, offering health forms in a patient's preferred language improves health insurance literacy rates. When Spanish speaking participants were given the same survey in both English and Spanish about their health insurance coverage, they had 56% and 80% correct responses, respectively [39]. It is crucial for providers serving children in Arizona and other locations with diverse populations to offer consistent language translation services to meet the community's needs and reduce access barriers amongst an already often underserved population.

Our results also found reduced acceptance of insurance plans (especially AHCCCS), which led to barriers in scheduling since we were unable to proceed without the requested insurance information. In instances where the insurance was not accepted, certain services were offered at high out-of-pocket costs, around $50 to $550, that may be unaffordable for many. Approximately 49% of Arizona children are covered by either AHCCCS or the state Children's Health Insurance Program, KidsCare, representing a significant amount of the overall pediatric population [43]. Refusing services to children on AHCCCS contributes to health disparities for this population who are already navigating negative social determinants of health, barriers to equal health, and educational opportunities. Previous studies have shown that providers may be less likely to accept Medicaid patients because the payment for services for these patients is reduced [41]–[45]. However, not providing care to AHCCCS recipients violates the providers' duty and social contract [40],[46]. In addition, patients experiencing high out of pocket costs, especially low-income families, are less likely to see and access care [47]. Practices, especially larger organizations, should explore options to negotiate lower cost-sharing options for patients and be mindful of the costs they list for basic and screening services for those paying out of pocket or with minimal insurance coverage. In turn, commercial insurance companies offering vision plans should ensure that these services are fully covered or at minimal cost to their enrollees.

These findings are consistent with previous studies that have shown limitations in overlap related to variation in provider availability and accepted insurance providers [18],[20]. Therefore, we recommend providers to accept all insurance carriers including AHCCCS to address these barriers, and take necessary steps to minimize out-of-pocket expenses or high co-pays for patients, especially for basic screening and vision care services.

## 6. Limitations

First, to obtain an accurate and working list of currently licensed and practicing clinicians in the state of Arizona, we contacted respective licensing boards for MD, DO, and OD vision care practitioners. We received updated (as of January 2022) licensee data from the Arizona Medical Board (MD) and the Arizona Board of Optometry (OD). However, the OD licensee data did not require licensees to provide office addresses, which resulted in an assortment of residential and office addresses. Efforts were taken to identify the correct office addresses and telephone numbers of these clinicians to obtain the most current and accurate look at the vision care landscape in Arizona. Furthermore, we attempted to contact the Arizona Osteopathic Board (DO) on several occasions via different routes but were ultimately unsuccessful in obtaining an updated list. Consequently, the list of osteopathic ophthalmologists included in this study were incorporated from a list current as of 2019.

Another limitation to note is the varied operating hours of each practice. As many of the practices operated during traditional business hours with shortened Fridays and varied lunch breaks, this restricted the times available to call the practices. It should also be emphasized that this study involved placing a direct phone call to each of the practices. If a practice was unreachable, we did not leave voicemails and followed up at a later date. The availability of online appointment scheduling systems also varied between practices. Further, it should be noted that while practices were often open during traditional business hours, the clinicians had hours that were different from those of the office operating hours.

Furthermore, due to the complexities in providing adequate information for the intended survey, Indian Health Service (IHS) and Veterans Affairs (VA) -operated facilities were excluded from the study except for one IHS contractor that provided information. Because IHS and VA facilities require unique identifiers of their patient population, e.g. Social Security numbers or service numbers, obtaining data from these providers was not feasible.

Finally, considering that this study was conducted in Arizona, the results may not be directly applicable to states with dissimilar characteristics.

## 7. Conclusion

Inadequate provider networks can hinder patients, especially children, from receiving care from the providers they know, trust, and depend upon throughout their lives. Regular screenings, eye exams and access to vision aids directly impact educational success and future employment opportunities- a crucial factor for all children and especially vital for those from low-income backgrounds, contributing to their ability to break free from cycles of poverty. Ensuring adequate, affordable, and accessible provider vision care networks align with the goal of holistic and whole-body care for both private and public health insurance recipients. Particularly for those on Medicaid, meeting our heightened duty to low-income and vulnerable children is critical, ensuring stewardship for this publicly funded safety net program. While the study focused on vision care access in Arizona, key findings and recommendations can be scaled to optimize children's vision care and outcomes, providing valuable insights for addressing network adequacy challenges in other regions.

## Use of AI tools declaration

The authors declare they have not used Artificial Intelligence (AI) tools in the creation of this article.


