# Supplementary material for: Evaluating the network adequacy of vision care services for children in Arizona: A cross sectional study
Source: AIMS Public Health. 2024 Jan 29;11(1):141–59. doi: 10.3934/publichealth.2024007 (PMC11007422; doi:10.3934/publichealth.2024007)
Supplement: Supplementary file 1 [file publichealth-11-01-007-s001.pdf]

Public Health

AIMS Public Health, 11(1): 141–159.
DOI: 10.3934/publichealth.2024007
Received: 28 September 2023
Revised: 09 January 2024
Accepted: 10 January 2024
Published: 29 January 2024

http://www.aimspress.com/journal/aimsph

Research article

Evaluating the network adequacy of vision care services for children in Arizona: A cross sectional study

Rizwana Biviji[1,*], Nikita Vora[2], Nalani Thomas[1], Daniel Sheridan[1], Cindy M. Reynolds[3], Faith Kyaruzi[1] and Swapna Reddy[1,*]

[1] College of Health Solutions, Arizona State University, Phoenix, AZ
[2] College of Arts and Sciences, Emory University, Atlanta, GA
[3] School of Medicine, University of Arizona, Phoenix, AZ

* **Correspondence:** Rizwana Biviji: Email: Rizwana.Biviji@asu.edu, Tel: +16024962318; Swapna Reddy: Email: Swapna.Reddy@asu.edu, Tel: +12547805284.

Supplementary

Survey Script

In calls conducted from May through June 2022, 5 Research Assistants (CR, NV, DS, FK, JO) followed this standardized set of guidelines outlined in the script below on behalf of a 10-year-old and 3-year-old patient covered either through an AHCCCS or commercial health insurance plan. The script was developed and approved as part of the research protocol filed through the ASU Institutional Review Board.

You are a "secret shopper", presenting on each call as the parent of a 10 and 3-year- old child, seeking to schedule a routine vision checkup. Calls should be conducted during normal business hours for each practice, which can be referenced on the provider spreadsheet. Before calling, you will need to determine what insurers each office contracts with. This information should be found on their website and will also be recorded on the provider spreadsheet.

- **North** (Mohave, Coconino, Yavapai, Navajo, and Apache)
    - **Indicated Health Plan: Care 1st**
- **Central** (Maricopa, Gila, and Pinal)
    - **Indicated Health Plan: Mercy Care**
- **South** (La Paz, Yuma, Pima, Santa Cruz, Cochise, Graham, and Greenlee)
    - **Indicated Health Plan: Banner University Family Care**

For commercial health insurance plans, insurers are categorized according to 2 service provider types:

- **Physicians** (MD, DO)
    - **Indicated Health Plan: Blue Cross Blue Shield of AZ**
- **Optometrists**
    - **Indicated Health Plan: Avesis**

Good morning/afternoon! My name is [FIRST/LAST NAME] and I'm calling on behalf of my daughter/son, _____________, to schedule a [Role A or Role B] as a new patient with Dr. _______

➔ *Age*

_____ *is 10 years old.*

➔ *Insurance #1: AHCCCS*

We have [PREDETERMINED AHCCCS PLAN]. Health plans should correspond to the GSA, as shown above.

Role A: I am seeking to schedule an eye checkup for my 10-year-old daughter as recommended by her pediatrician. She seems to be having some vision trouble and may need glasses. (Follow through setting up an appointment).

You are on Medicaid (AHCCCS).

Also, what ages do you serve? I may need to set up a routine eye care visit for my younger child as well. (If asked for the age of the younger child, respond- 3-year- old).

Role B: I am seeking to schedule an eye checkup for my 10-year-old son as recommended by his pediatrician. He seems to be having some vision trouble and may need glasses. (Follow through setting up an appointment).

You are on a predetermined commercial health insurance plan.

Also, what ages do you serve? I may need to set up a routine eye care visit for my younger child as well. (If asked for the age of the younger child, respond- 3-year- old).

1. Could I schedule an appointment with Dr. _______________?

[If "yes" or "yes, but you need a referral"—continue with (2) below]

[If "no"—continue with B1]

2. When is Dr. _______________'s next opening?
3. When is the next availability after [1st date provided]?
4. Would my child need to see another clinician before seeing a specialist (MD/DO)?

[If "no"—continue with (5) below]

[If "yes"—continue with C1]

5. (When calling as an AHCCCS recipient:) Can I get an appointment if I can only pay through AHCCCS?

[If "yes"—continue with (6) below]

[If "no"—continue with (R1)]

6. Also, is Dr. ________________ bilingual? (If asked which language you prefer, respond- Spanish)
7. Does he/she offer after hours (morning/evening) or weekend appointments?

If/when you are offered an appointment, do not finalize, or confirm. End the call with the following:

Perfect. I will just need to give you a call back later today/tomorrow after I confirm my work schedule. Thank you so much for your time!

After the call has ended, make sure that you have recorded all information in the spreadsheet.

B Module—If Program Cannot Schedule and Appointment

(B1) List reasons why appointment cannot be scheduled (check all that apply)

1. Not currently scheduling ophthalmologists or optometrists (MD/DO/OD)—none are available *[go to (R1)]*
2. Only schedule appointments for urgent or emergent conditions *[go to (R1)]*
3. Only schedule appointment for other eye care professionals, not ophthalmologists (MD/DO) *[go to (C1)]*
4. Other ____

C Module—If Patient Needs to See another Clinician before Seeing an Ophthalmologist

(C1) When is the next available appointment with this other clinician? *[Record exact date and time; then return to main survey and continue with (6)] [If respondent cannot access appointment system without real information, ask, "About how long is the wait for such an appointment?"]*

(C2) And after that appointment, how long would the wait be to see an Ophthalmologist? *[Record response].*

Referral Module—Where Program Would Refer People they couldn't Schedule

(R1) So if I couldn't get an appointment with this provider, is there somewhere else you could refer me? Do you have a phone number?

[Record name and contact information] [Later, follow up with calls to referred offices]

This script has been adapted from the works of Steinman et al. [1] and Reddy et al. [2].

Reference:

1. Steinman KJ, Kelleher K, Dembe AE, et al. (2012) The use of a "mystery shopper" methodology to evaluate children's access to psychiatric services. *J Behav Health Serv Res* 39: 305–313. https://doi.org/10.1007/s11414-012-9275-1

2. Reddy S, Speer M, Saxon M, et al. (2021) Evaluating network adequacy of oral health services for children on Medicaid in Arizona. *AIMS Public Health* 9: 53–61. https://doi.org/10.3934/publichealth.2022005

AIMS Press

© 2024 the Author(s), licensee AIMS Press. This is an open access article distributed under the terms of the Creative Commons Attribution License (http://creativecommons.org/licenses/by/4.0)
